# Unveiling the role of epigenetic mechanisms and redox signaling in alleviating multiple abiotic stress in plants

**DOI:** 10.3389/fpls.2024.1456414

**Published:** 2024-09-19

**Authors:** Surbhi Shriti, Anirban Bhar, Amit Roy

**Affiliations:** ^1^ Division of Plant Biology, Bose Institute, Kolkata, West Bengal, India; ^2^ Post Graduate Department of Botany, Ramakrishna Mission Vivekananda Centenary College (Autonomous), Rahara, Kolkata, India; ^3^ Faculty of Forestry and Wood Sciences, Czech University of Life Sciences, Prague, Czechia

**Keywords:** abiotic stress, crop resilience, epigenetic regulation, histone modification, reactive oxygen species, anthropogenic disturbances

## Abstract

Anthropogenic activities and subsequent global climate change instigate drastic crop productivity and yield changes. These changes comprise a rise in the number and severity of plant stress factors, which can arise simultaneously or sequentially. When abiotic stress factors are combined, their impact on plants is more substantial than that of a singleton stress factor. One such impact is the alteration of redox cellular homeostasis, which, in turn, can regulate downstream stress-responsive gene expression and resistance response. The epigenetic regulation of gene expression in response to varied stress factors is an interesting phenomenon, which, conversely, can be stable and heritable. The epigenetic control in plants in response to abiotic stress combinations and their interactions with cellular redox alteration is an emerging field to commemorate crop yield management under climate change. The article highlights the integration of the redox signaling pathways and epigenetic regulations as pivotal components in the complex network of plant responses against multi-combinatorial stresses across time and space. This review aims to lay the foundation for developing novel approaches to mitigate the impact of environmental stresses on crop productivity, bridging the gap between theoretical understanding and practical solutions in the face of a changing climate and anthropogenic disturbances.

## 1 Introduction

The collective influence of human activities on Earth over the last few decades has resulted in numerous severe environmental stress conditions within our ecosystems and agricultural areas (Rillig et al., 2019). These conditions encompass extreme and variable weather events attributable to climate change, such as heatwaves, frost, prolonged submergence, or drought. Additionally, they affect soil conditions, including salinity and varying pH, and they introduce various anthropogenic contaminants like heavy metals, microplastics, pesticides, antibiotics, and persistent organic pollutants into the environment. Other contributors to the complex environmental milieu include radiation (e.g., UV), restricted nutrient availability, and elevated concentrations of airborne molecules and gases such as ozone, particulate matter from combustion, and carbon dioxide (CO_2_).

In agriculture, the impact of these simultaneous stress conditions creates crop damage of different magnitudes. Plants often encounter a combination of abiotic stresses in their natural habitats, creating a dynamic and challenging environment that leads to a loss in crop productivity and yield. Optimizing crop yield is a paramount objective directly proportional to the formidable challenges posed by these stressors (Zhang et al., 2022). Abiotic stress, arising from non-living environmental factors, includes a spectrum of adversities such as extreme temperatures, drought, salinity, and pollutants. Each of these stressors, individually and collectively, profoundly influences plant physiological processes, thereby precipitating a discernible reduction in crop productivity. Drought conditions may coincide with elevated temperatures, salinity stress may accompany heavy metal toxicity, and fluctuating environmental factors may converge, presenting a complex matrix of challenges. Numerous reports and studies indicate the ill effects of individual stress factors. However, our knowledge of simultaneous stress remains rudimentary. Therefore, understanding how plants perceive, prioritize, and respond to these multi-combinatorial stresses at the molecular level requires a holistic approach. Multi-combinatorial stress denotes the simultaneous exposure of cells to various environmental challenges, necessitating sophisticated and adaptive cellular responses for endurance (Zandalinas and Mittler, 2022).

Redox signaling, characterized by the production and reception of reactive oxygen species (ROS), stands at the nexus of plant stress responses. The alteration of redox homoeostasis is considered the primary and essential cellular response against any stress. Once considered mere byproducts of cellular metabolism, ROS is now recognized as versatile signaling molecules that modulate various cellular processes. The integration of redox signaling in stress perception allows plants to sense and transduce signals in a dynamic and context-dependent manner, initiating a cascade of events that culminate in adaptive responses. In the context of cellular homeostasis, the interaction between epigenetic regulatory mechanisms and redox signaling has emerged as a focal point of investigation, particularly when confronted with multicombinatorial stressors simultaneously. Epigenetic control, characterized by heritable modifications to DNA, histones, and non-coding RNAs, orchestrates gene expression patterns without altering the underlying genomic sequence. Concurrently, redox signaling involves maintaining the delicate equilibrium between ROS and antioxidants, indispensable mediators in cellular signaling pathways. The amalgamation of these regulatory frameworks assumes heightened significance when cells are subjected to a confluence of environmental stressors, precipitating situation-dependent cellular response (Ueda and Seki, 2020).

The experimental work of Zandalinas et al. (2021) has introduced a crucial concept in plant biology: as the number and complexity of stressors on a plant increase, plant growth and survival decline significantly, even when each individual stressor is relatively mild. This synergistic effect of multifactorial stress was previously observed in soil microbiomes by Rillig et al. (2019), highlighting its potential impact on plant–microbiome interactions. In ecosystems with high biodiversity (3D forest ecosystems), outcomes of multifactorial stress can vary, but the effect is likely negative in low-biodiversity agroecosystems like crop fields. The plant’s resistance response to multifactorial stress is intricately linked to redox signaling and epigenetic regulation. Reactive oxygen species (ROS) play a critical role in signaling pathways that modulate stress responses, while epigenetic mechanisms regulate gene expression patterns without altering the DNA sequence. Understanding how these processes interact under combined stress conditions is essential for developing resilient crops. The review underscore the urgent need to limit the number and intensity of environmental stressors to prevent detrimental impacts on plant health and ecosystem stability. The initial observations by Rillig and Zandalinas call for further studies on multifactorial stress combinations across various plant species, microbiomes, and crops, with a focus on the roles of ROS and epigenetics. Addressing this issue requires a comprehensive approach that includes breeding and engineering plants for resilience, increasing crop diversity, and manipulating plant–microbiome interactions. Integrating laboratory and field experiments with genome-wide association studies and leveraging wild plant varieties and microbiomes can enhance crop resilience. Additionally, incorporating insights from material sciences, nanotechnology, physics, chemistry, and precision agriculture can help mitigate the effects of multifactorial stress. Despite the challenges posed by the increasing rate of anthropogenic activity, with concerted efforts, it is possible to develop strategies to counteract the negative impacts of multifactorial stress on crops and ecosystems, particularly through advancements in understanding ROS signaling and epigenetic modifications. Understanding the integration of redox signaling pathways and epigenetic control in plant responses to multiple abiotic stresses holds profound implications for crop improvement strategies (Suzuki et al., 2014). Unravelling the molecular underpinnings aids in developing targeted interventions to enhance stress tolerance and resilience in agriculturally important crops, ultimately contributing to global food security. In summary, this review explores the integration of the redox signaling pathways and epigenetic regulations as a central node in the complex web of plant responses to multiple abiotic stresses. By deciphering the molecular dialogues within this complex network, we aim to extend our knowledge of plant stress biology and pave the way for innovative strategies to bolster crop resilience in a changing world.

## 2 ROS and its cellular toxicity

**Figure 1:**
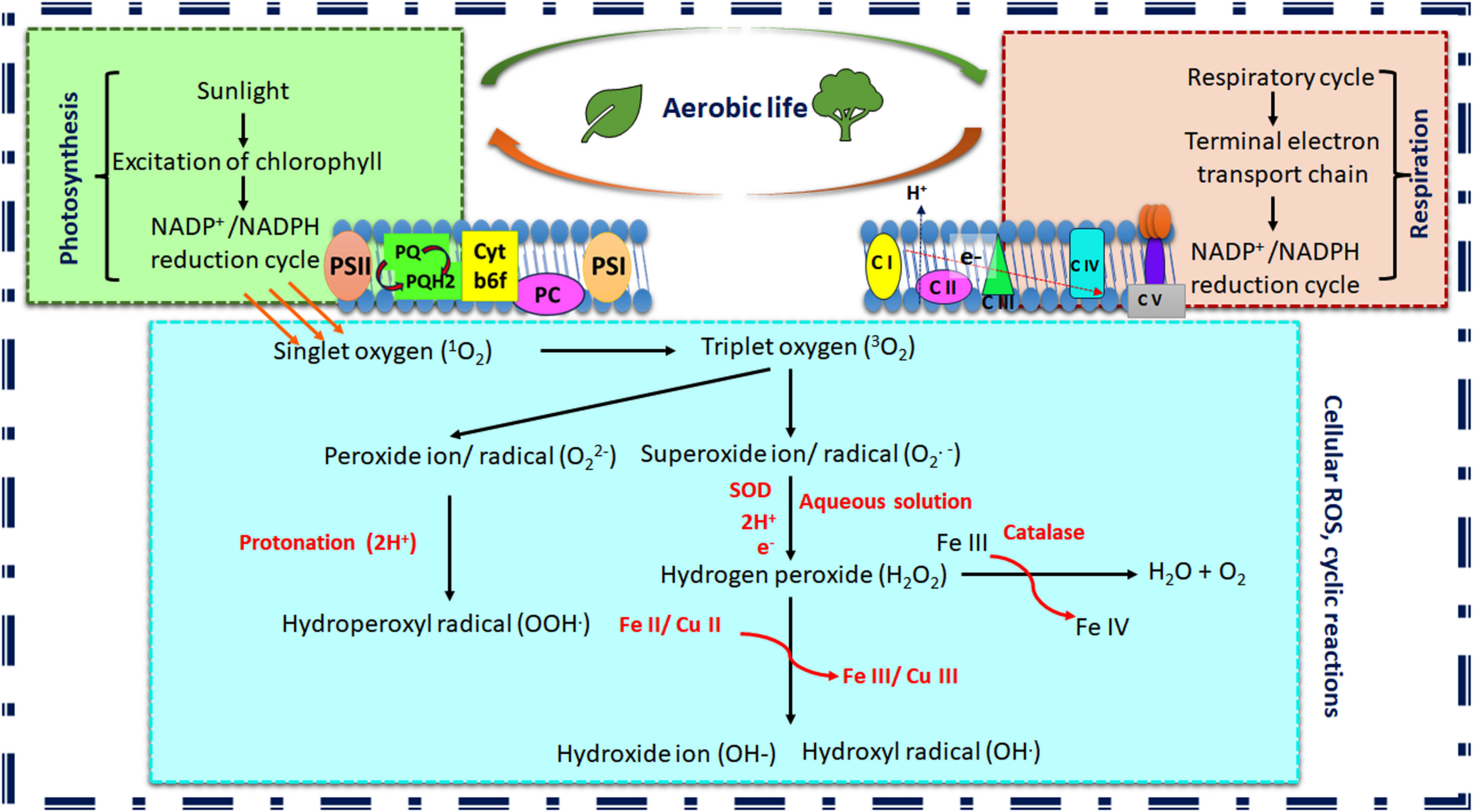
Schematic representation of cyclic reactions leading to reactive oxygen species (ROS) generation in plants. The figure depicts the apparent ROS production due to aerobic life. Photosynthesis and respiration are the primary biochemical cycles in plants that generate ROS as byproducts (PSI, PhotosystemI; PSII, PhotosystemII; PQ, Plastoquinone; Cyt-b6f, Cytochrome b6f complex; PC, Plastocyanine; CI-CV, Cytochrome I-V; SOD, Superoxide dismutase; NADPH, Nicotinamide Adenine Dinucleotide Phosphate Hydrogen.

ROS and reactive nitrogen species (RNS) are the most abundant active signaling intermediate in the cellular milieu. The molecules containing one or more lone pair of electrons are called free radicals. Oxygen and nitrogen are two vital molecules necessary for sustaining life and the generation of essential biomolecules. O_2_ can carry two lone pair of electrons, whereas N_2_ can bear three. The generation of active oxygen species (AOS) is common in aerobic life forms. ROS includes both radical and non-radical forms of active oxygen molecules generated through partial reduction of O_2_, e.g., oxygen radical or superoxide anion or superoxide radical (O_2_^•–^), hydroxyl radical (HO^•^) and hydrogen peroxide (H_2_O_2_), hydroxide ion (HO^−^), peroxide ion (O_2_^−2^) (Ray et al., 2012). H_2_O_2_ is not as reactive as the other reactive forms; it is generated in the chain reaction process. This category has also included the spontaneous generation of H_2_O_2_ from superoxide radicals. The radical form is more reactive due to reactive lone pairs and high reduction potential (Baek and Skinner, 2012). The triplet oxygen (O_2_^2•^) and sometimes singlet oxygen (^1^O_2_) are also considered ROS (Pospíšil et al., 2019). The mystery behind the generation of active oxygen lies in its spin chemistry of lone pairs of electrons. The spin quantum numbers of this pair of electrons are the same; hence, it restricts the acceptance of electrons from another atom with similar spin. The oxidation of molecular O_2_ is only possible through one electron transfer to another paramagnetic center, usually transition metals (Fe and Cu) with unpaired electrons (Tripathy and Oelmüller, 2012). The source-to-sink transition of ROS relies on chain reactions depending on the redox potential of the elements (Pospíšil et al., 2019; Mansoor et al., 2022). The light-activated NADP^+^/NADPH system during photosynthesis in chloroplast and NAD^+^/NADH system during electron transport system in chloroplast can donate electrons to the molecular oxygen due to high reduction potential (-320 mV). The light-driven photoactivation of chlorophyll molecules into singlet and triplet forms is also parallel induction during photosynthesis. In this process, both peroxide ion (O_2_^−2^) and superoxide radicals (O_2_^•−^) are formed within plants. Gradually the antioxidant scavenger system, including superoxide dismutase (SOD) with an iron core and free iron (FeIII) plays a crucial role in a further reduction to form hydrogen peroxide and/or peroxide radicals (H_2_O_2_/HO•) before it is further reduced to O_2_ or water (Tripathy and Oelmüller, 2012; Pospíšil et al., 2019; Mansoor et al., 2022) (**Figure 1**).

Although highly reactive and toxic when accumulated, ROS are vital for normal plant growth, development, and stress signaling (Huang et al., 2019). The toxicity of ROS depends upon their longer half-life and extended diffusion capability within the cell (Mittler, 2017; Waszczak et al., 2018). The superoxide radicals (O_2_^. −^) are usually produced in mitochondrial ETS and can affect the mitochondrial genome. For instance, the light-grown maize seedlings showed high mitochondrial DNA damage due to excessive ROS production in developing mitochondria from non-pigmented meristematic cells (Tripathi et al., 2020). Singlet oxygen has almost similar transmission capability through the plasma membrane and has a greater affinity towards Trp, His, Tyr, and Cys residues of proteins (Dumanović et al., 2021). They can be transmitted from the chloroplast membrane to the cytosol and accumulate within the cell. The site-specific accumulation leads to programmed cell death (PCD) or hypersensitive response (HR) mediated cell death or stress response against varied abiotic factors (Dmitrieva et al., 2020). As the scavenging enzyme system of singlet oxygen is lacking, overproduction of this may be deleterious for the survivability of the plants. The delimited production of singlet oxygen may be involved in regulated cell death, controlling the normal development of plants (Bhatt et al., 2021). Recently, it has been observed that singlet oxygen governs pivotal signaling in the degradation of damaged chloroplasts (Lemke and Woodson, 2022). The mutant analysis with *Arabidopsis fluorescent* (flu) showed that SAFEGUARD1 (SAFE1), a chloroplast granum localized protein, is involved in the protection of chloroplast grana membrane in EXECUTER1 (EX1)-dependent retrograde signaling pathway (Wang et al., 2020). Recently, it has been demonstrated that EXECUTER 2 helps the EX1 signalosome to sense singlet oxygen in plastids (Dogra et al., 2022). The H_2_O_2,_ instead, has a greater half-life and transmission time, hence targeting Cys and Met residues of proteins away from their origin. H_2_O_2,_ after being produced by the activity of SOD in the chloroplast, mitochondria, plasma membrane NADPH oxidases, peroxisomal oxidases, type III peroxidases, etc., are transported within the cell by aquaporins. The accumulation of H_2_O_2_ leads to many toxic effects in plants but are readily detoxified by catalases, peroxiredoxin, glutathione peroxidases (GPX), and ascorbate peroxidases (APX) (Bhar et al., 2017). The accumulation of H_2_O_2_ may also lead to autophagy and PCD (Smirnoff and Arnaud, 2019). *Fusarium*-induced redox signaling was also observed in chickpeas during wilt disease. The susceptible plants showed extensive oxidative damage and membrane degradation due to poor antioxidative scavenging efficiency compared to the resistant chickpea plants (Gupta et al., 2013). Highly reactive HO• can modify almost all biomolecules (DNA, RNA, lipids, and proteins) in the cellular vicinity (Dumanović et al., 2021). The biotic and abiotic factors are responsible for the induction of ROS and target the biomolecules through varied transcription factors, e.g., NAC, Zinc finger, WRKY, ERF, MYB, DREB, and bZIP (Khedia et al., 2019). In most cases, ROS are accumulated and cause oxidative bursts in response to stress factors. Phenolic acids play critical roles in scavenging these heavy metal-induced ROS in *Kandelia obovata* and increase the bioavailability of metals (Chen et al., 2020). Soil aluminum (Al) induces oxidative bursts, interfering with plant water and nutrient uptake by affecting several nutrient uptake and aquaporin gene families (Chauhan et al., 2021). It has been concluded that uncontrolled production of ROS due to inefficient or damaged scavenging machinery leads to oxidative damage that includes degradation of biomolecules, membrane damage, disruption of cellular permeability, modification of metabolic enzymes, reduced carbon fixation, yield loss and cell death. However, balanced production of ROS is necessary for the normal functioning of plant cells. Hence, the toxicity of ROS is intricately dependent upon its cellular detoxification efficiency.

## 3 Plant immunity, ROS metabolism, and regulation

**Figure 2:**
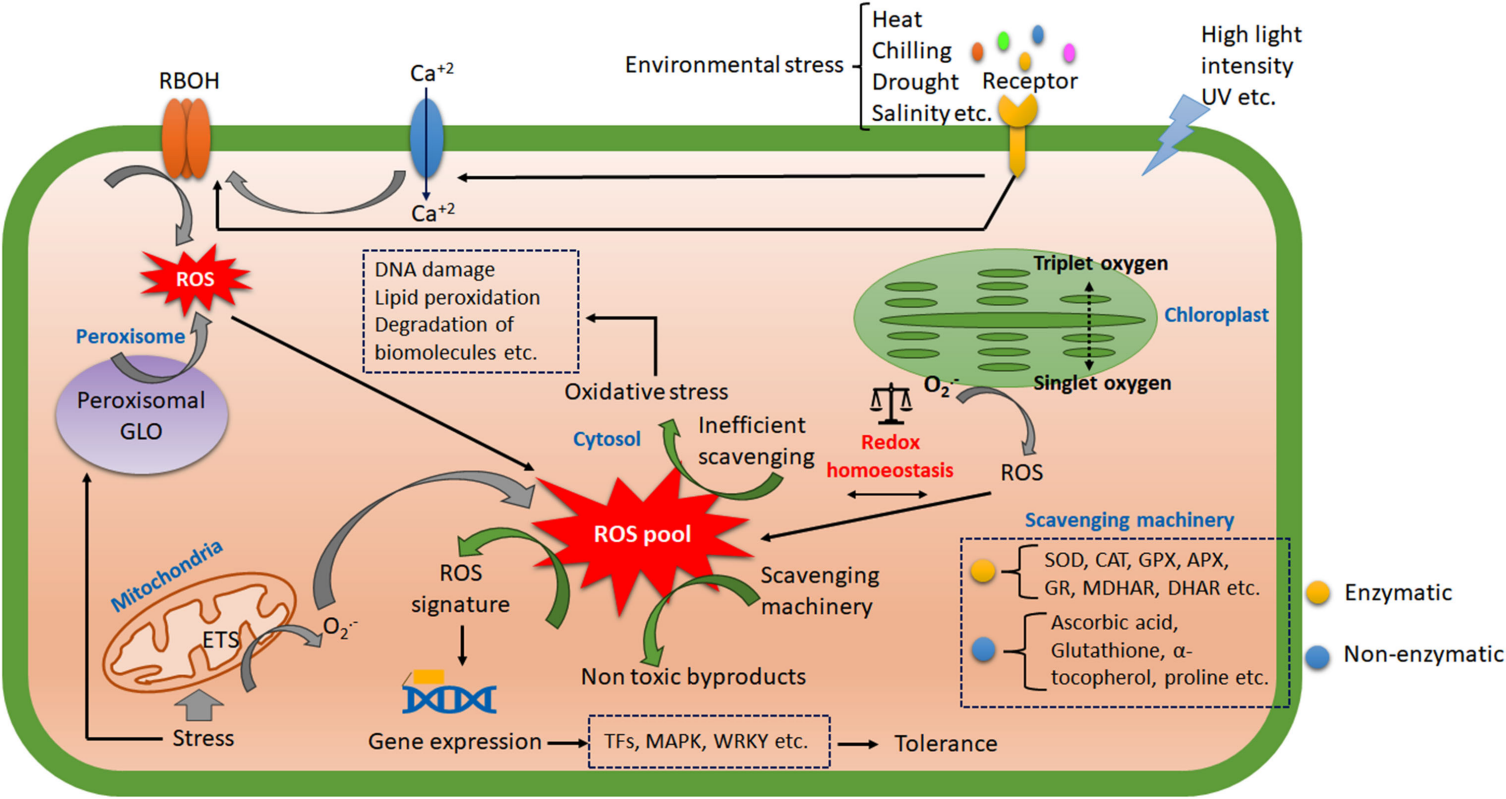
The overview of ROS signal transduction during abiotic stress. The receptors in the cell membrane perceive the major abiotic stress and transduce signals to several membrane-bound transporters. The calcium influx plays a crucial inducer of respiratory burst oxidase (RBOH) to generate ROS. The organelles also contribute to the intra-cellular ROS pool. The activity of enzymatic and non-enzymatic scavengers has balanced the cellular redox homoeostasis. The efficient scavenging produces a ROS signal that activates varying transcription factors and is responsible for stress-induced gene expression. On the other hand, inefficient scavenging leads to oxidative stress and macromolecular damage.

The plant immune response and signaling rely on biotic interaction. The pathogen-associated molecular pattern (PAMP) and host pattern recognition receptors (PRR) interact to instigate the first line of the immune response, pattern-triggered immunity (PTI). In the second line, the most specific and robust immune signaling takes place through the interaction of pathogen-secreted effector molecules (toxins) with the host resistance (R) gene, called effector-triggered immunity (ETI) (Jones and Dangle, 2006). The induction of ROS is an inevitable reaction in both PTI and ETI with varied magnitude (Yuan et al., 2021). This immune response may sometimes be impregnated as the genetic imprint and transgenerational, termed immunogenic memory (Bhar et al., 2021). The abiotic stress response does not always follow a generalized path, but oxidative burst is a common physiological effect that affects almost every abiotic stress factor. The host cells have specific receptors for abiotic stress signals. Membrane-bound receptor-like kinases (RLKs) play crucial roles in perceiving external stress signals in plants (Osakabe et al., 2013). Many biotic stress receptors may also act as abiotic stress receptors. Usually, the membrane damage associated with fluidity, integrity and the cell wall degradation product in response to both biotic and abiotic stress produces intermediates that act as damage/danger-associated molecular patterns (DAMPs). These DAMPs regulate many cellular responses, gene expressions, and hormonal cross-talk (Saijo and Loo, 2020). The receptor-mediated stress signals activated membrane-bound respiratory burst oxidase homolog (RBOH) or plant nicotinamide adenine dinucleotide phosphate oxidases (NADPH oxidases) (Liu and He, 2016). The receptor-mediated signal sometimes instigates calcium influx channels and generates a calcium signature, activating RBOH homologs (Bhar et al., 2023). The light-induced generation of singlet and triplet oxygen-mediated ROS production has also contributed to cellular redox homeostasis (Li and Kim, 2023). Despite their photodamaging behavior, they are also involved in cytosolic ROS signaling. Mitochondria, conversely, produces superoxide radicals during improper electron transport within the mitochondrial membrane. The abiotic stress and climate change induce elevated metabolism and produce proliferous ROS subsequently within mitochondria (Nadarajah, 2020). Alternatively, peroxisomal glycolate oxidases (GLO) play crucial roles in peroxisomal ROS production (Corpas, 2019). Climate change-induced heat stress and other abiotic stress combinations were reported to induce GLO-dependent H_2_O_2_ production in rice with elevated photosynthetic activity (Balfagón et al., 2020). The antioxidative scavenging machinery in plants actively detoxifies these ROS. The imbalanced production and scavenging system or inefficient detoxification led to oxidative stress in plants. The enzymatic scavengers include superoxide dismutase (SOD), catalase (CAT), ascorbate peroxidase (APX), glutathione peroxidase (GPX), monodehydroascorbate reductases (MDHAR), dehydroascorbate reductases (DHAR), glutathione reductase (GR) etc. The non-enzymatic scavengers encompass carotenoids, α-tocopherol, glutathione (GSH), ascorbic acid (AsA) and proline, which act on ROS to maintain cellular redox state (Mansoor et al., 2022). Thus, when the metabolic and stress-induced ROS surpasses the scavenging system, an oxidative burst takes place. Multiple abiotic stress stringently regulates acclimation using these redox alterations (Choudhury et al., 2017). The ROS leads to DNA damage, membrane degradation, lipid peroxidation etc. as oxidative stress markers (Bhar et al., 2023). Balanced ROS may act as a signaling intermediate and coordinates resistance gene expression by multiple induction of transcription factors (MYB, bZIP, WRKY, RAV, NAC, AP2/ERF, ZAT etc.) and *cis* acting elements (ARE, CORE, W-box, GCC box, as-1 like etc (Singh et al., 2019) (**Figure 2**).

**Figure 3:**
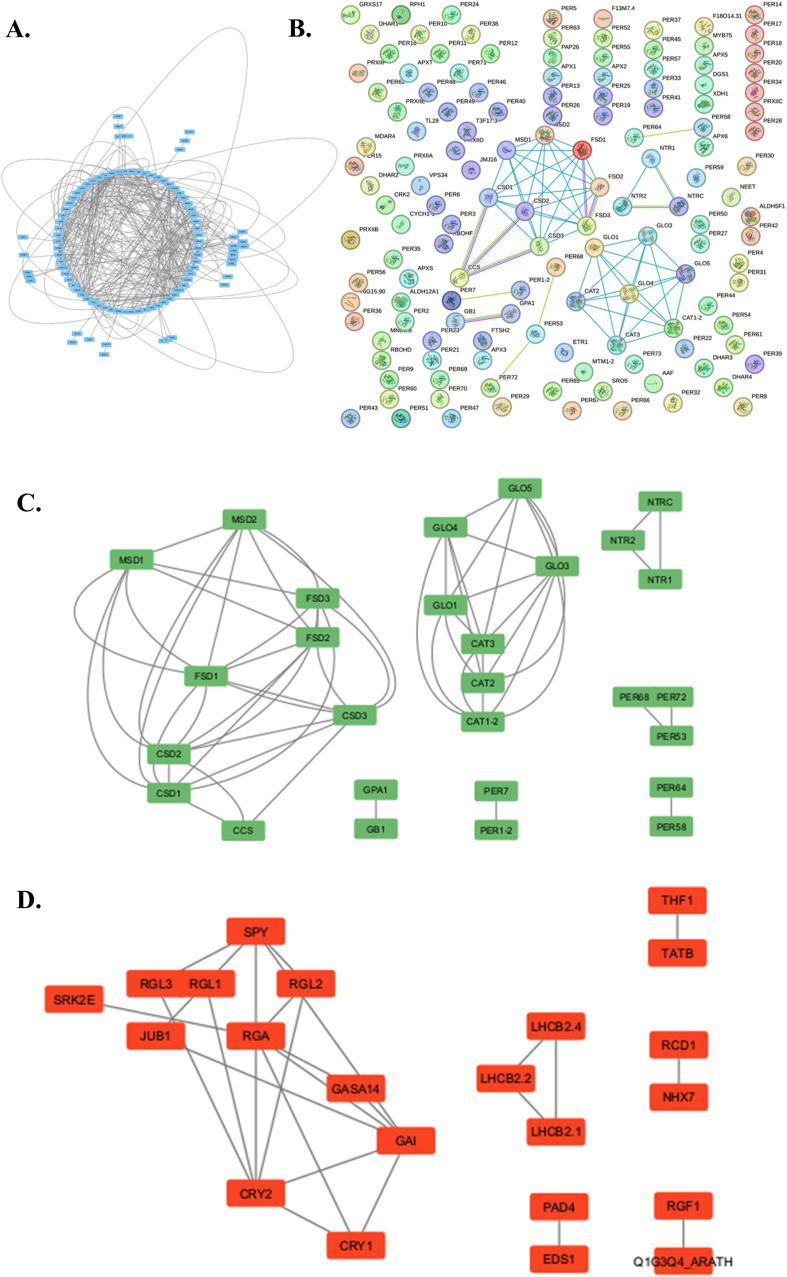
The metabolic network showing ROS-related regulation and metabolism in *Arabidopsis thaliana* analyzed by STRING (12.0) and further simplified and visualized through Cytoscape (3.10.1). **(A)** The entire ROS interactome illustrates intricate metabolic interactions. **(B)** The physical subnetwork analysis as visualized in STRING (12.0). **(C)** The simplified subnetwork shows the physical interaction of metabolic processes involving ROS (Cytoscape 3.10.1). **(D)** The simplified subnetwork demonstrates ROS metabolism regulation during stress (Cytoscape 3.10.1).

The network analysis of *Arabidopsis* ROS-regulated genes using STRING 12.0 (https://string-db.org/) (Szklarczyk et al., 2023) has revealed a considerable interactome consisting of 138 nodes and 395 edges with the PPI enrichment p-value, < 1.0e-16. The physical interaction-dependent subnetworks revealed clusters of genes involved in ROS metabolism. SOD homologs, mainly Mn-SOD, Fe SOD, and Cu-Zn SOD (MSD, CSD, FSD) produce an interacting cluster. Peroxisomal GLO produces another metabolic cluster with CATs. Thioredoxin reductase (NTRs) and peroxidases (PERs) produce independent metabolic networks. The regulation of ROS metabolism under abiotic stress demonstrated that gibberellic acid (GA) plays a critical role in *Arabidopsis*. Many GA-related genes and DELLA repressor-like proteins (GA, GASA, GAI, RGL, RGA) produce a common cluster with cytochrome-dependent proteins (CRY1, CRY 2). The transcription factor JUB1 (JUNGBRUNNEN), a negative leaf senescence regulator, interacts directly with the GA cluster. In addition, JUB1 modulates cellular H_2_O_2_ levels and enhances tolerance to various abiotic stresses by regulating DREB2A (Xu et al., 2020). JUB 1 interacts with SPINDLY (SPY). The SPY is an O-linked N-acetylglucosamine transferase (OGT) involved in various processes, such as the gibberellin (GA) signaling pathway and circadian clock. OGTs catalyze the addition of nucleotide-activated sugars directly onto the polypeptide through O-glycosidic linkage with the hydroxyl of serine or threonine. It probably acts by adding O-linked sugars to yet-unknown proteins. OGTs act as a repressor of the GA signaling pathway to inhibit hypocotyl elongation (Qin et al., 2011). Light-harvesting complexes (LHCB) regulate light-driven ROS generation, forming separate network clusters. Lipase-like phytoalexin deficient 4 (PAD4); required downstream of MPK4 pathway for accumulation of the plant defense-potentiating molecule, salicylic acid. The PAD4 is also considered the significant ROS regulator in *Arabidopsis* and enhanced disease susceptibility 1 (EDS1). Sodium/hydrogen exchanger (NHX), thylakoid formation 1 (THF), Sec-independent protein translocase protein (TATB), and Serine/threonine-protein kinase (SRK2E) are found to be other regulators of ROS metabolism in *Arabidopsis* (**Figure 3**; **Supplementary Tables 1, 2**).

## 4 Sources of ROS production and its signaling during abiotic stress

As discussed earlier, ROS play a dual role in biological systems, serving as essential signaling molecules and potential mediators of oxidative damage. This largely depends on its production vs. scavenging efficiency. In the context of abiotic stress, a plethora of environmental factors, such as extreme temperatures, drought, salinity, and pollutants, can perturb cellular homeostasis, leading to an upsurge in ROS production. Hence, understanding the balance between ROS generation and signaling during abiotic stress is pivotal for deciphering the molecular mechanisms underlying plant stress responses. In plants, cellular ROS production is multifaceted as diverse sources contribute to their generation under abiotic stress conditions. From mitochondria and chloroplasts to peroxisomes and apoplast, each cellular compartment has distinct machinery for ROS production (Dietzel et al., 2008; Gilroy et al., 2016; Huang et al., 2016; Kerchev et al., 2016; Rodríguez-Serrano et al., 2016; Takagi et al., 2016). Elucidating the complex signaling pathways that transduce ROS-generated signals is critical for deciphering the adaptive strategies employed by organisms to cope with environmental challenges is essential. Furthermore, the redox status of individual ROS-producing organelles and cumulative cellular redox status will vary according to the stress condition faced by the plant. Different environmental factors, stress combinations, and permutations will result in distinct intracellular redox state subsets and a unique signature stress response.

### 4.1 Abiotic stress-induced ROS production and signaling in chloroplasts

The green plants bear chloroplast as a unique organelle for photosynthesis. These chloroplasts play a significant role in generating ROS, serving as crucial signals in the cross-talk between chloroplasts and the nucleus. The photosynthetic electron transport chain’s (PETC) evolution is designed to control reactive oxygen species (ROS) generation, limit accidental injury, and support critical signaling pathways under abiotic stress conditions. In chloroplast, the abiotic stress, which constricts the availability of carbon dioxide by the closure of stomata, increases the production of ROS such as H_2_O_2,_ O_2_^·-^ and ^1^O_2_. This, in turn, disturbs the redox status, leading to the initiation of retrograde and anterograde signaling (Asada, 2006; Gill and Tuteja, 2010; Baniulis et al., 2013; Kleine and Leister, 2016; Mignolet-Spruyt et al., 2016). The stoichiometry of photosystems is also affected by stress-induced ROS production as the distribution of energy balance between PSI (Photosystem I) and PSII (Photosystem II) is altered (Dietzel et al., 2008; Vainonen et al., 2008; Pesaresi et al., 2009). The antioxidant systems in the thylakoid and stromal regions control the collection of ROS originating from photosynthesis, thereby governing environmental perception by adjusting oxidative signals exported from chloroplasts to the nucleus (Fichman et al., 2019; Gollan and Aro, 2020).

ROS form an integral part of a network of retrograde signals that modulate chloroplast functions based on prevailing metabolic and environmental conditions during organelle biogenesis and photosynthesis. Several chloroplast-to-nucleus signals, including those triggered by the accumulation of 3’-phosphoadenosine 5’-phosphate (Estavillo et al., 2011), methylerythritol cyclodiphosphate (Bjornson et al., 2017), dihydroxyacetone phosphate (Vogel et al., 2014), or heme (Espinas et al., 2012), have been identified. 3’-Phosphoadenosine 5’-phosphate, a product of sulfur metabolism, is generated through the transfer of the sulfate group from phosphoadenosine phosphosulphate (PAPS) by sulphotransferases, impacting molecules like desulphoglucosinolates and salicylic acid (Kopriva and Gigolashvili, 2016). The SAL1/FRY1 phosphatase prevents the accumulation of 3’-phosphoadenosine 5’-phosphate by degrading it to inorganic phosphate (Pi) and adenosine monophosphate (AMP) within chloroplasts and mitochondria (Chan et al., 2013, 2016). Under stress conditions, oxidative inactivation of SAL1 leads to increased 3’-phosphoadenosine 5’-phosphate accumulation. This pathway connects mitochondrion-to-nucleus and chloroplast-to-nucleus signaling, regulating transcription factors ANAC013 and ANAC017, which mediate a ROS-related retrograde signal from mitochondrial complex III (Shapiguzov et al., 2019). ANAC013 and ANAC017 functions are suppressed by the nuclear-localized RADICAL-INDUCED CELL DEATH1 (RCD1) protein, acting as a hub linking chloroplast and mitochondrial signaling to control metabolism in both organelles.

Chloroplast antioxidants, such as stromal ascorbate peroxidase (sAPX) and thylakoid ascorbate peroxidase (tAPX), may regulate retrograde signaling in plant responses to environmental stresses. tAPX, localized mainly in unstacked regions of the thylakoid membrane, efficiently removes H_2_O_2_ produced at PSI at the expense of ascorbate (Maruta et al., 2012). Ascorbate regeneration occurs at the thylakoid membrane and chloroplast stroma. tAPX, with higher turnover rates for H_2_O_2_ than peroxiredoxin (PRX), protects PSII more effectively from photooxidative damage (Dietz, 2016). The role of the glutathione (GSH) pool and dehydroascorbate reductases (DHARs) in regenerating ascorbate and detoxifying ROS is under scrutiny (Rahantaniaina et al., 2017). Nevertheless, recent research suggests GSH involvement in ascorbate recycling under high-light conditions (Terai et al., 2020). Production of these also curbs the ill effects of excessive ROS in chloroplast (Mittler et al., 2004). The production of ROS radicals also leads to nuclear reprogramming of gene expression, which leads to programmed cell death and chlorosis as a phenotype. It also activates a considerable subset of stress-related responsive genes. These gene expressions are executed by two chloroplast proteins associated with thylakoid membranes known as EXECUTER1 and EXECUTER2, both being transcribed in the nucleus (Wagner et al., 2004; Lee et al., 2007; Kleine and Leister, 2016). The accumulation of ROS during abiotic stress is managed by enzymes that can scavenge ROS and specific pathways like Fe- and Cu Zn-SODs and the Asada–Foyer–Halliwell pathway.

### 4.2 Abiotic stress-induced ROS production and signaling in mitochondria

Metabolism is the primary target of any type of stress, biotic or abiotic. The flow of electrons across the Electron Transport Chain (ETC) and during oxidative respiration generates ROS, including the superoxide ion and hydrogen peroxide (Moller, 2001; Murphy, 2009). The primary locations for generating ROS are ETC complexes I, II, and III (Andreyev et al., 2015). The ETC consistently produces superoxide and hydrogen peroxide in normal conditions called metabolic ROS. The various alterations in metabolism and stress-related conditions can induce a more reduced state in the ETC, resulting in heightened production of ROS, called stress-induced ROS. This phenomenon occurs, for example, when the ETC is inhibited or slowed down, particularly when stress reduces the availability of ADP. Consequently, respiratory inhibitors can impede ETC complexes, causing over-reduction in different segments of the ETC and resulting in excess superoxide production (Schwarzländer et al., 2009). Changes in mitochondrial ultrastructure triggered by stress, such as those observed in salt stress, have the potential to directly impact or impair the functioning of ETC and Oxidative Phosphorylation (OXPHOS) (Garcia de la Garma et al., 2015).

Particularly in response to abiotic stresses, the production of mitochondrial ROS increases significantly. Stress conditions disrupt the normal functioning of the respiratory electron transport chain in mitochondria, leading to the leakage of electrons from complex I and III and the formation of superoxide radicals (O_2_^•-^), which can be further converted to H_2_O_2_ by Mn-SOD (Quan et al., 2008; Huang et al., 2016). While superoxide has a brief half-life, the longer half-life of hydrogen peroxide allows it to traverse membranes through aquaporins, potentially functioning as a signaling molecule that connects mitochondria to other cellular compartments. The dynamic involvement of ROS from mitochondria has been connected to ROS signaling mechanisms (Ng et al., 2014). These mechanisms encompass oxidized intermediates, Ca^2+^ ions, and phosphorylation processes. Additionally, they are associated with transcriptional and post-transcriptional modifications in cell functioning, which can regulate growth and development, including programmed cell death (PCD) (Singh et al., 2016; Cui et al., 2019). Alternative oxidase (AOX), type II NAD(P)H dehydrogenase, and uncoupling proteins in the inner mitochondrial membrane can all help to slow down this process (Noctor et al., 2007; Rasmusson and Wallström, 2010). WRK15 binds to the promoter region of AOX1 to regulate the production of reactive oxygen species (Vanderauwera et al., 2012). Also, retrograde signaling between mitochondria and the nucleus is activated by altering ROS production in mitochondria during abiotic stress (Woodson and Chory, 2008).

Interestingly, mitochondrial redox alterations are also sometimes connected to hormonal imbalances. ROS signaling in mitochondria and the signaling of mitochondrial disturbances are connected to developmental processes controlled by auxins. *Arabidopsis* FtsH4 mutants, characterized by a deficiency in a mitochondrial protein-processing protease, exhibit an accumulation of mitochondrial hydrogen peroxide linked to the disruption of auxin homeostasis, dysregulation of the cell cycle, and impairment of meristem activity (Dolzblasz et al., 2018). The mutation of the splicing factor ABA-overly-sensitive-8 (ABO8), crucial for the accurate expression of the NAD4 component of complex I, leads to heightened production of mitochondrial ROS, increased sensitivity to abscisic acid (ABA), diminished auxin accumulation/signaling, and reduced meristem activity (Yang et al., 2014). The interaction among mitochondrial ROS, hormones, and developmental processes suggests that mitochondrial ROS signaling might be influenced, to some extent, by pathways associated with hormone signaling.

### 4.3 Abiotic stress-induced ROS production and signaling in peroxisomes

Peroxisomes are cellular organelles involved in various metabolic processes, including the breakdown of fatty acids and purines. During abiotic stress, peroxisomes can contribute to ROS production due to the activity of enzymes involved in these metabolic pathways, such as fatty acid beta-oxidation. Increased photo respiration causes increased hydrogen peroxide production by the enzyme glycolate oxidase in peroxisomes (Foyer and Noctor, 2009; Tripathy and Oelmüller, 2012; Kerchev et al., 2016). The antioxidant enzyme catalase (CAT) alleviates this over-accumulation of photo-respiratory reactive oxygen species. Hydrogen peroxide signaling during stress conditions is majorly understood by studying mutants deficient in peroxisomal CAT (Kerchev et al., 2016). Like other organelles, peroxisomal ROS causes cellular redox status changes and gene transcription in the nucleus (Vanderauwera et al., 2005).

There are at least four different processes through which ROS production in abiotic stress is mediated. Among these, NADPH Oxidases (Respiratory Burst Oxidase Homologs - RBOHs) are the most widely studied mechanism (Gilroy et al., 2014, 2016). This mechanism links ROS signaling and calcium signaling during stress and the production of superoxide molecules in the apoplast. RBOHs deficient mutants, for instance, RBOHD and RBOHF, for studying the interplay between redox status and abiotic stress tolerance. Hence, RBOHs have been shown to play an essential role in signaling mechanisms that assist in plant tolerance against abiotic stresses. Peroxidases are another enzyme mediating ROS generation in peroxisomes (O’Brien et al., 2012). Peroxidase-dependent ROS generation is shown to be involved in potassium deficiency response and regulation of root growth (Kim et al., 2010). Oxalate oxidase is another enzyme produced in peroxisome that regulates hydrogen peroxide production in the cell of roots and is shown as an essential protein in drought strengths (Voothuluru and Sharp, 2013). Ma et al. (2016) proposed another enzyme known as xanthine dehydrogenase that plays a vital role in stress signaling. Mitigation of ROS levels in apoplast is conducted by Cu/Zn-SODs, APXs, cell wall-bound peroxidases, and low levels of ascorbate and GSH. Compared to intracellular ROS production, apoplastic ROS production is ineffective, leading to imbalance. This disparity is a critical event in the signaling system and stress response.

## 5 ROS signaling in simultaneous abiotic stresses

Numerous research studies have been conducted to evaluate plant response against singular abiotic stress conditions. However, abiotic stress generally occurs in combination rather than independently in reality. Stress response at molecular, physiological, and metabolic levels tends to vary significantly in combinatorial abiotic stress compared to individual stress. Combinatorial abiotic stress in plants refers to the simultaneous occurrence of multiple stress factors that can negatively impact plant growth and development. These stress factors can include two or more abiotic stressors, creating a complex and challenging environment for plants (**Table 1**). Combinatorial stress response causes a major loss in crop productivity and yield (Mittler, 2006; Mittler and Blumwald, 2010; Suzuki et al., 2014). As a result, combination stressors are now the subject of more laboratory research since they are more damaging and have a greater detrimental effect on crop output. Additionally, distinct transcriptome signatures corresponding to combinations of abiotic stressors—for example, heat stress coupled with salinity or drought—are observed. These signatures comprise many transcripts that differ significantly from those of individual stress responses (Rizhsky et al., 2004). Besides, contrary to popular belief, some stress combinations even provide some amount of tolerance to another stress (abiotic or biotic) when it occurs after them.

**Table 1:** List of studies that attempted to unravel simultaneous stress impacts on plants.

Stress 1	Stress 2	Species	Type of Stress	References
**Temperature**	Waterlogging	*Brassica oleracea*	Heat	(Lin et al., 2015)
		*Gossypium hirsutum L.*	Heat	(Chen et al., 2017)
		*Arabidopsis thaliana*	Cold	(Xu et al., 2019)
		*Triticum aestivum*	Cold	(Li et al., 2014)
	Drought	*Arabidopsis thaliana* Pad2.1	Cold	(Kumar et al., 2015)
		*Gossypium hirsutum* L.	Heat	(Zafar et al., 2023)
		Winter Barley	Heat	(Jampoh et al., 2023)
		*Arabidopsis thaliana*	Heat	(Wolfe and Tonsor, 2014)
		*Hordeum vulgare L.*	Heat	(Rollins et al., 2013)
		*Triticum aestivum*	Heat	(Szucs et al., 2010)
		Soybean	Heat	(Rahman et al., 2023)
		Chickpea	Heat	(Nayyar et al., 2014; Benali et al., 2023)
		*Sorghum bicolor*	Heat	(Johnson et al., 2014)
		*Camellia sinensis*	Cold	(Zheng et al., 2016)
		Tomato	Cold	(Zhou et al., 2020)
		*Elymus nutans* Griseb	Cold	(Liu et al., 2023)
		*Vitis amurensis and V. vinifera* cv. *‘Muscat Hamburg’*	Cold	(Su et al., 2015)
		Creeping Bentgrass	Cold	(Zhang et al., 2015)
		Wheat	Cold	(Li et al., 2014)
	Salinity	*Chenopodium quinoa*	Heat	(Becker et al., 2017, p. 201)
		*Brachypodium dystachion*	Drought + Heat	(Shaar-Moshe et al., 2017)
		*Arabidopsis thaliana*	ABA + Heat	(Prasch and Sonnewald, 2013; Suzuki et al., 2016)
		*Jatropha curcas*	Heat	(Silva et al., 2013)
		Alfalfa	Acid precipitation and Cold	(Bao et al., 2020)
	Heavy Metal	*Wheat*	Zinc Heat/Cold	(Suszek-Łopatka et al., 2021)
		Wheat	Yttrium and Heat	(Gong et al., 2022)
	UV Radiation	*Capsicum annuum*	Cold	(León-Chan et al., 2017)
		Cotton	Carbon dioxide	(Brand et al., 2016)
		Maize	Fluctuating Ambient Temperature	(Singh et al., 2014)
	Light	Tomato	Heat	(Rivero et al., 2014)
		Tomato	Cold	(Shu et al., 2016)
		Wheat	Heat	(Chen et al., 2017, p. 207)
		*Myrica rubra Sieb. et Zucc.*	Heat	(Gao et al., 2019)
		*Persea americana Mill* cv. *‘Hass’*	Cold	(Joshi et al., 2020, p. 201)
		Tomato	Heat	(Lu et al., 2017; Zhou et al., 2020)
**Waterlogging**	Salinity	*Porteresia coarctata*		(Garg et al., 2014)
	Heavy Metal	*Cynodon dactylon*		(Tan et al., 2017)
**Drought**	Salinity	*Pisum sativum* L.		(Attia et al., 2020)
		Canola		(Sharif et al., 2018, p. 20)
		Wheat		(Dugasa et al., 2019)
		Spinach		(Ors and Suarez, 2017)
		Barley		(Osthoff et al., 2019)
		Tibetan Barley		(Ahmed et al., 2013)
		*Festuca arundinacea* Schreb.		(Esmailpourmoghadam et al., 2023)
		*Brachypodium dystachion*	Triple Stress (Heat)	(Shaar-Moshe et al., 2017)
	Heavy Metal	Grape	Silicon and Potassium	(Haddad and Kamangar, 2015)
		*Phaseolus vulgaris* L	Cadmium	(Yildirim et al., 2023)
		*Hymenaea stigonocarpa*	High Light	(Costa et al., 2015)
		*Carica papaya* L	High Light	(Vincent et al., 2018)
**Salinity**	Heavy Metal	Broccoli	Boron	(Lin et al., 2015)
		*Cajanus cajan (L.)*	Cadmium	(Garg and Chandel, 2012)
	UV Radiation	Rosemary		(Hamidi-Moghaddam et al., 2019)
	Gamma Radiation	*Zea mays* L		(Aliyeva et al., 2023)
	Light	Hydrocotyle vulgaris		(Samsone et al., 2020)
**Heavy Metal**	UV Radiation	Soybean	Cadmium	(Esmailpourmoghadam et al., 2023)
**Ozone**	Salinity	Wheat		(Zheng et al., 2014)
	Temperature	*Brassica juncea* L	Heat	(Lee et al., 2020)
	UV Radiation	*Linum usitatissimum L.*		(Tripathi and Agrawal, 2013)
		Sunflower		(Tripathi et al., 2019)

From the redox point of view, much evidence indicates differences in ROS levels, gene expression of various ROS enzymes, and antioxidant systems when compared between combinatorial abiotic stress and individual stress application. Quantities of O^2.-^, H_2_O_2_, lipid peroxidation byproducts, expression of enzymes like SOD, APX, CAT, AOX, peroxidases, glutathione-S-transferase, glutathione reductase, and GPX, and accumulation of antioxidants like ascorbate, GSH, flavonols, phenolic compounds, alkaloids, tocopherol, and carotenoids, as well as osmo-protectants like proline, glycine betaine, trehalose, and sucrose, indicated these changes. Their expression patterns were observed to be distinct in response to multiple stressors. A specific redox status is likely a unique signature to abiotic stress combinations within the confinement of exclusive physiological responses to combinatorial stresses. The paramount example is to open the stomata to cool off and close it to avoid loss of excess water by the plants. However, seeing how these pressures interact with a plant will be intriguing (Rizhsky et al., 2002, 2004). Another interesting study by Koussevitzky et al., 2008 showed that *Arabidopsis* cytosolic APX1 (apx1) deficient mutants were more sensitive to drought heat stress than thylakoid APX, suggesting that hydrogen peroxide levels in the chloroplast are more critical for tolerance against this specific combinatorial stress. Similarly, *Arabidopsis* mutant deficit in ABA and ROS-regulator protein called PP2Cs were highly sensitive to salinity and heat stress as well as drought and heat stress, indicating the significance of interactions between abscisic acid and ROS signaling, which in turn is very important for plant stress tolerance (Suzuki et al., 2016). Based on research studies conducted on stress combination in plants, it was proposed that it is an important node in stress acclimation in differentially regulated ROS-responsive transcripts, as reviewed by Suzuki et al., 2014. The redox alteration is directly involved in the hypersensitive response mediated cell death in many cases (Liu and Zhang, 2021). Alternatively, it is also involved in downstream signaling. It has been found that in the case of the heat stress response (HSR), ROS are involved in the activation of different heat shock factors (HSFs) and heat shock proteins (HSPs) (Fortunato et al., 2023). In these signaling events, plant thiol peroxidases play critical roles in sensing and transducing the abiotic stress signals (Vogelsang and Dietz, 2022). Along with the induction of redox-mediated transcription factors (TFs), it is also involved in the epigenetic control of gene expression. It has been studied extensively in animals, and it was found that nuclear factor erythroid 2-related factor 2 (NRF2) is involved in this signal transition and epigenetic control. It is an emerging field in plant science research, and many interesting findings have been documented in recent years, comprehensively discussed in the following section.

## 6 Abiotic stress-mediated redox alteration: a trigger for epigenetic changes

Plants employ a sophisticated repertoire of molecular responses to navigate the complexities of abiotic stress. Central to this ensemble is the complex interaction between redox signaling and epigenetic modifications. This section provides an overview of the fundamental concepts, emphasizing the importance of studying the cross-talk between redox and epigenetic pathways. Epigenetic modifications are stable inheritable changes in gene expression that are not encoded in the DNA nucleotide sequence. It primarily includes DNA methylation, histone modifications, chromatin remodeling, and histone variants in plants. These changes can affect gene availability and activity, thus regulating many molecular functions, including gene transcription, DNA repair, and recombination. They are essential in controlling plant response to external stimuli, including abiotic stress. Since these modifications are reversible, they are controlled by various signals caused by developmental, environmental, and phytohormonal cues. The change in the redox status of cells caused by abiotic stress is highly significant among these signals. Abiotic stress triggers the production of ROS and disrupts cellular redox balance. Post-translational modification of gene product determines the fate of the final gene expression, which mediates the epigenetic pathway when introduced by oxidative stress. Considering the importance of such events, the following section explores the nuanced role of redox signaling in orchestrating stress responses, including the activation of defense mechanisms and the modulation of cellular processes. The nexus between redox changes and epigenetic modifications and how oxidative stress acts as a molecular trigger, influencing DNA methylation dynamics, histone modifications, and small RNA pathways, will also be discussed briefly.

### 6.1 DNA methylation-redox dynamics

Methylation and demethylation are interchangeable processes fundamental to epigenetic changes leading to altered gene expressions. Accumulation of evidence suggests that the presence of redox intermediates directs DNA methylome, which, in turn, regulates gene transcription. Generally, ROS accumulation leads to DNA hypomethylation, as confirmed by different studies. A comparative gene analysis study of *Arabidopsis* and its redox-compromised state transition 7 (stn7) mutant indicates that many nuclear genes were not expressed in their mutant form when treated with high light (Dietzel et al., 2008). However, its wild type had activated nuclear histone acetyltransferase (HAT) and histone deacetylase (HDAC), enzymes known to promote histone methylation and demethylation from the redox signals from their chloroplast.

Choi and Sano (2007) proposed that plants partially tolerate environmental stresses through epigenetic modifications. In their study, they found the activation of enzyme glycerophos- phodiesterase -like (NtGPDL) proteins in tobacco cells facing increased oxidative stress caused by the exposure of different inducers like paraquat, aluminum, cold stress, or salinity stress. Genomic loci of *NtGPDL* gene study, when further analyzed by methylation pattern study by bisulfite mapping, indicated selective demethylation of CG site of ORF of *NtGPDL.* The promoter region of the gene was also found to be demethylated. In another study, tobacco suspension culture, when treated with Juglone (5-hydroxy-1,4 naphthoquinone), showed increased over-accumulation of ROS species and subsequent hypo methylation of DNA (Poborilova et al., 2015). Similarly, Berglund (2017) also reported increased hypo methylation of DNA in *Pisum sativum* suspension culture when treated with nicotinamide (a precursor of NAD), known as redox intermediate. There was also an increase in the production of GSH. Although there are lots of studies in the animal kingdom regarding GSH-induced epigenetic changes, research remains scarce on plants. Ou et al. (2015) reported increased demethylation in rice seedlings treated with a NO donor such as sodium nitroprusside. It was seen that increased accumulation of ROS species by irradiation also causes epigenetic changes. A differential pattern of DNA methylation was observed in the aerial parts of *Arabidopsis* when exposed to irradiation concomitant with ROS accumulation (Ramakrishnan et al., 2022).

### 6.2 Histone acetylation-redox dynamics

**Figure 4:**
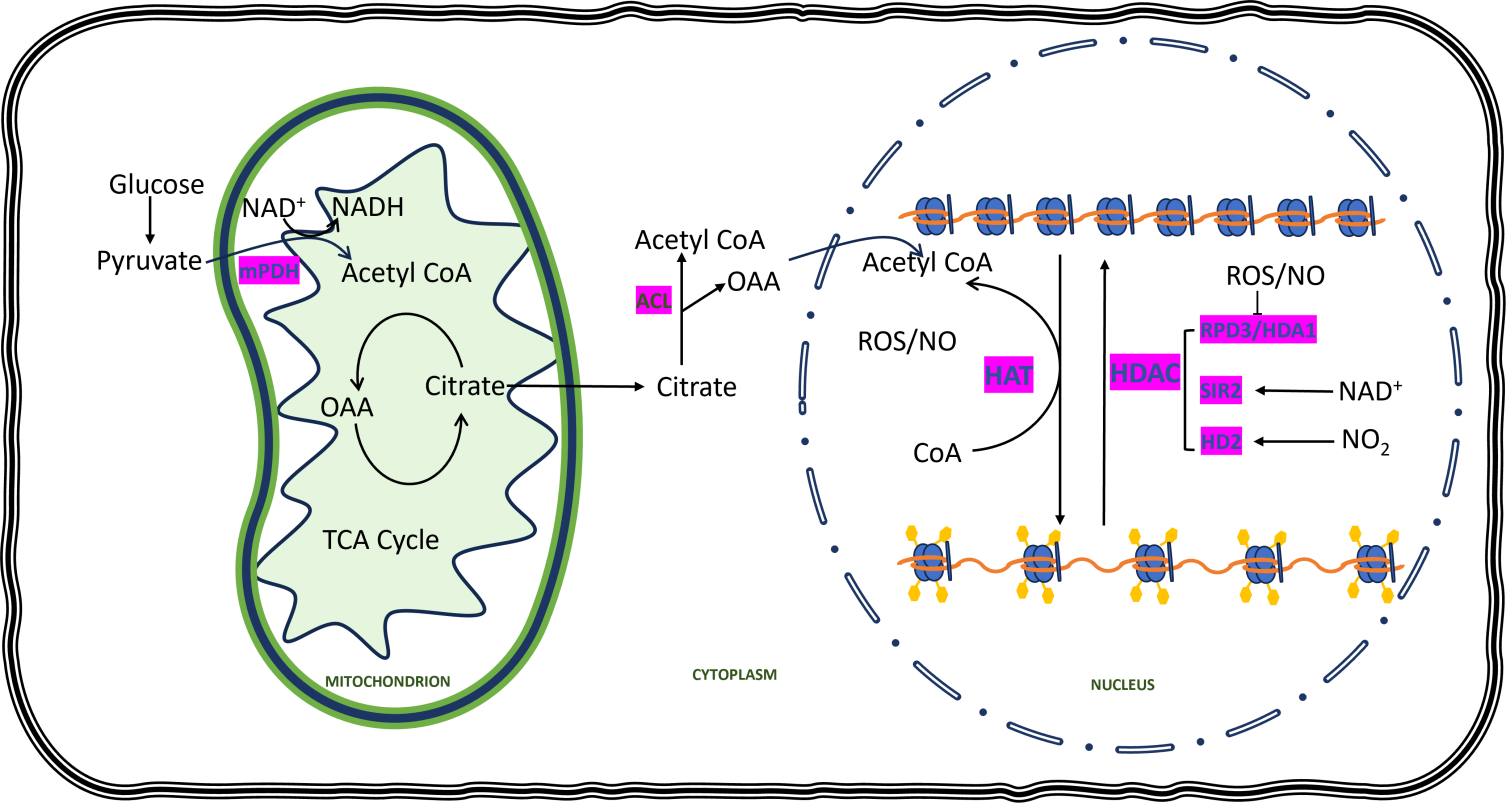
Redox components exert an influence on histone acetylation. In the cytoplasm, glucose undergoes breakdown to pyruvate, which enters the mitochondria and undergoes conversion to acetyl CoA by mitochondrial pyruvate dehydrogenase (mPDH), utilizing NAD+ for reduction. Acetyl CoA combines with oxaloacetate (OAA) generated in the TCA cycle to form citrate, which then enters the cytoplasm. Within the cytoplasm, citrate is converted back to OAA and acetyl CoA through ATP-citrate lyase (ACL). Acetyl CoA synthesized in the cytoplasm subsequently enters the nucleus, serving as the supplier of acetyl groups for the histone acetylation process. Histone acetyltransferases (HATs) utilize the acetyl group from acetyl CoA to introduce acetylation marks (Ac), yellow pentagons on the lysine residues of the histone tail, thereby weakening the interaction between DNA and histone and promoting gene expression. On the other hand, histone deacetylases (HDACs) remove histone acetyl groups, leading to chromatin compaction. Various HAT and HDAC enzymes are influenced by reactive oxygen species (ROS), nitric oxide (NO), and nicotinamide adenine dinucleotide (NAD+).

Acetylation of histone is another type of post-translational modification that occurs in the nuclear region. However, unlike methylation, histone acetylation usually assists in the expression of genes. Conversely, there are few reports of DNA methylation acting as promoters of gene expressions. The fate of histone acetylation depends on the presence of a substrate known as acetyl CoA, which is managed by the opposing activity of two enzymes known as HAT and HDAC. These enzymes are also redox-regulated. There is abundant research indicating the mediation of histone acetylation by the redox status of cells in plants and animals. Mounting evidence indicates that redox components regulate histone acetylation by influencing the accumulation of acetyl CoA. It has been elucidated that the conversion of pyruvate to acetyl CoA is facilitated by the pyruvate dehydrogenase (PDH) complex, wherein NAD^+^ serves as a cofactor for its catalytic activity (**Figure 4**). In maize seedlings, caused by heat induction led to increased accumulation of ROS which further increases the hyperacetylation by increased expression of HAT. Causevic et al., 2006 reported different levels of expression of reactive oxygen species directly related to different levels of histone acetylation.

As mentioned earlier, HAT enzymes play an important role in epigenetic regulation, and activity is affected by oxidative stress. In combination with other factors, HAT enzyme forms are complex, and its activity is affected by oxidative stress. A component of the HAT complex known as elongator protein is reported to mediate anthocyanin biosynthesis and oxidative stress in *Arabidopsis* (Zhou et al., 2009). The induction of salinity stress on maize seedlings led to increased electrolyte leakage, which activated the antioxidant pathway genes (AbdElgawad et al., 2016). Additionally, there was increased production of gene expression of HAT enzymes Such as *ZmHATB* and *ZmGCN5* and upregulation of cell wall synthesis genes*, ZmEXPB2* (expansin-B2) and *ZmXET* (xyloglucan endotransglycosylase). Upon further examination, it was found that this accumulation was mediated by increased acetylation of their promoter region. This proves that the HAT enzyme mediates different stress responsive genes by altering their acetylation under stress.

### 6.3 Histone deacetylation-redox dynamics

As discussed earlier, gene expression increases with the higher acetylation level. Hence, stress-induced gene modulation primarily functions with the decreased action of histone deacetylase enzymes. Non-sirtuins [non- (SIRTs)] are a group of HDACs that regulate histone acetylation in cells. This becomes unrecruited from the repression of genes under oxidative stress. This action further drives increased acetylation and increased gene expression. In rice seedlings, it was reported that upon successful host-pathogen interactions, the transcript level of *HDT701* was increased (Ding et al., 2012). When overexpressed in rice, this increases susceptibility to various abiotic stresses, including salinity and osmotic stress and decreased H4 acetylation. When the same gene was silenced, the whole pattern was reversed with increased expression of defense-related genes and increased H4 acetylation. Increased and enhanced acetylation marks on the promoter region of defense-related genes are generally linked to their increased expression. Non-sirtuins HDACs are sensitive to redox potential, and their production is connected to the presence or absence of oxidative stress. Under oxidative stress, these HDACs get inactivated, leading to the acetylation of histones in the promoter regions of stress-responsive genes, leading to their increased production and acclimation to abiotic stress tolerance (Luo et al., 2017). Many HDACs have been reported to possess redox switches for better oxidative stress tolerance (Jänsch et al., 2020).

Similarly, in *Arabidopsis*, cold stress induces degradation of HD2C by increasing acetylation levels of histone H3, which is promoted by CULLIN4-based ubiquitin E3 ligase. This increased acetylation activates COR or cold-responsive genes (Park et al., 2018). It further interacts with HDAC to modulate acetylation level and respective stress tolerance regulation. In double mutant *Arabidopsis*, HD2C directly interacted with HDA6, causing decreased demethylation of histone H3K9 and increased histone H3K9K14 acetylation. This activates ABA-responsive genes. Also, HD2C further interacts with the BRM -SWI/SNF chromatin remodeling complex, thereby regulating *Arabidopsis* response to heat stress (Luo et al., 2012).

## 7 Mechanism of redox and epigenetic alterations due to multiple abiotic stress responses in plants

While adaptive responses to single stress factors have been extensively studied under controlled laboratory conditions across various species (e.g (Ritonga and Chen, 2020; Takahashi et al., 2020; Zhao et al., 2020; Jethva et al., 2022; Zhang et al., 2022), research on the mechanisms underlying adaptation to multiple stresses in natural environments, such as combinatorial stress, remains limited. This gap in understanding is particularly evident in the context of flooding, where the molecular responses to simultaneous or sequential stresses, such as salinity or drought-induced soil conditions, are still not well-defined despite the frequent occurrence of these combined stressors in nature (Yin et al., 2023).

Flooding often coincides with other environmental stresses such as salinity, temperature extremes, and heavy metals, leading to complex interactions that can be antagonistic, additive, or synergistic. These interactions trigger a cellular energy crisis and activate transcriptional reprogramming through transcription factor families like ANAC and ERFVII. These factors, modulated by proteins like kinases, help integrate multiple stress responses, particularly in managing reactive oxygen species (ROS). The unique physiological responses resulting from these stress combinations often involve distinct gene and epigenetic regulation, with key signaling components like ERFVII and RBOHD playing central roles in coordinating the plant’s response to multiple stresses (Renziehausen et al., 2024).

Flooding challenges plants by causing oxygen deficiency, leading to hypoxia or anoxia, which is worsened by ethylene entrapment and reduced light during submergence (Voesenek and Bailey-Serres, 2015; Sasidharan et al., 2017). In response to hypoxia, plants undergo significant transcriptomic and metabolic changes, shifting from aerobic respiration to anaerobic glycolysis due to limited O_2_ availability, which substantially reduces energy production. Reactive oxygen species (ROS) signals, generated by both the mitochondrial electron transport chain (mtETC) and RESPIRATORY BURST OXIDASE HOMOLOG (RBOH) activity, play a critical role in hypoxic adaptation, triggering systemic responses to waterlogging (Fukao and Bailey-Serres, 2004; Hong et al., 2020). Transcription factors like ANAC013 and ANAC017 are central to mitochondrial retrograde signaling (MRS) and submergence tolerance, regulating key genes that mitigate ROS damage and enhance low-oxygen resilience (Bui et al., 2020; Eysholdt-Derzsó et al., 2023). Disruption of these pathways leads to excessive ROS accumulation, reduced anoxia tolerance, and impaired photosynthetic efficiency during reoxygenation (Van Aken et al., 2016; Jethva et al., 2023).

By decoupling mitochondrial electron transport chain (mtETC) activity from ATP production, UNCOUPLING PROTEIN 1 (UCP1) limits excessive reactive oxygen species (ROS) formation, thereby preventing over-reduction of mtETC proteins (Barreto et al., 2014, 2017, 2022). Although mitochondrial retrograde signaling (MRS) primarily relies on ROS signals, UCP1 enhances hypoxia-induced transcriptional changes, including the induction of ALTERNATIVE OXIDASE 1a (AOX1a). This effect is likely linked to UCP1’s role in stabilizing ETHYLENE RESPONSE FACTOR VII (ERFVII) transcription factors (TFs) by inhibiting the N-degron pathway, which otherwise marks proteins for degradation (]. In Arabidopsis, the ERFVII subfamily, comprising five members—RELATED TO APETALA 2.2 (RAP2.2), RAP2.3, RAP2.12, HYPOXIA RESPONSIVE ERF 1 (HRE1), and HRE2—plays a crucial role in low-oxygen sensing and subsequent gene regulation (Nakano et al., 2006). These TFs bind to the HYPOXIA-RESPONSIVE PROMOTER ELEMENT (HRPE) found in approximately 70% of hypoxia core gene (HCG) promoters, initiating transcriptional responses under hypoxic conditions (Zubrycka et al., 2023). RAP2.12, for instance, regulates the expression of RESPIRATORY BURST OXIDASE HOMOLOG D (RBOHD) and HYPOXIA RESPONSIVE UNIVERSAL STRESS PROTEIN 1 (HRU1), which together mediate ROS production under hypoxia (Gonzali et al., 2015; Yao et al., 2017).

ERFVII proteins possess a conserved cysteine residue in their N-termini, making them susceptible to degradation through the Arg/Cys branch of the N-degron pathway in an oxygen (O_2_)-and nitric oxide (NO)-dependent manner This degradation process involves sequential modifications: methionine is first removed by METHIONINE AMINOPEPTIDASES (MetAPs), followed by O2-dependent cysteine oxidation by PLANT CYSTEINE OXIDASES (PCOs), and then arginylation by ARGINYL TRANSFERASE ENZYME 1 (ATE1) and ATE2, which tags the TF for proteasomal degradation via PROTEOLYSIS 6 (PRT6) (Weits et al., 2014; White et al., 2017; Masson et al., 2019). However, in lowland rice, the ERFVII factor SUBMERGENCE 1A (SUB1A) is crucial for survival under flash flooding, as it escapes this degradation pathway by interacting its N-terminus with its C-terminus. Moreover, ethylene stabilizes ERFVII proteins during submergence by inducing PHYTOGLOBIN 1 (PGB1), a NO scavenger that prevents ERFVII degradation (Hartman et al., 2019). RAP2.12, specifically, avoids degradation under normal oxygen conditions by associating with ACYL-COA BINDING PROTEIN 1 (ACBP1) and ACBP2 at the plasma membrane and, upon hypoxia, dissociates to accumulate in the nucleus, thus contributing to the hypoxia response (Licausi et al., 2011; Kosmacz et al., 2015; Schmidt et al., 2018).

The combination of differential regulation of different redox sensors mentioned above and its role in epigenetic regulation to combat multicombinatorial stress is still naive. However, rapid acceleration in research to address the knowledge gaps in this field of study is also critical for our understanding.

## 8 Cross-talks between redox pathway and epigenetic regulations during multiple abiotic stress

Alteration of the cellular redox state is inevitable in response to any abiotic stress factor. The multiple abiotic stress impacts cellular redox state more dramatically. As discussed earlier, cellular redox homeostasis is contributed by the complex interaction between chloroplast-mitochondra-nucleus (Locato et al., 2018). The cellular antioxidative system efficiently contributes to modulating this homeostasis. The efficiency of these antioxidative systems determines ROS-induced hypersensitive response (ROS HR) or ROS-induced signaling in response to stress (Gupta et al., 2013). The redox state also directly controls the gene expression by DNA methylation, histone acetylation, and histone methylation. The perception of stress depends upon the signal that originates from chloroplasts and mitochondria and is transmitted to the nucleus. The antagonistic and synergistic effect regulates the controlled expression of genes in response to multiple abiotic stress (Locato et al., 2018). The major redox-regulated proteins and non-proteins e.g., NADPH, glutathione, gluteredoxin, peroxiredoxins, ascorbate, thioredoxin, ferredoxin etc. participate in the generation of redox signatures, which in turn also controls the priming against multiple abiotic stress (Tripathi et al., 2024) This also triggers the site-directed epigenetic modification that also restrains the concurrent stress situations in plants significantly (Harris et al., 2023). The epigenetic imprints may also be involved in transgenerational memory development and resistance phenomenon in subsequent generations (Bhar et al., 2021). Substantial supportive studies are required to understand the exact mechanism of plant transgenerational memory development. The redox alteration also modulates the metabolism in plants in terms of the deregulation of several proteins and metabolite synthesis (Shen et al., 2016). This regulation may involve an anterograde trafficking system from the nucleus to the cytosol and then mitochondria and chloroplast. The primary signal of this retrograde signaling is ROS. The chloroplast and mitochondrial genome have a significant level of rearrangements during the course of different abiotic stresses (Dobrogojski et al., 2020; Meng et al., 2022). More research is necessary to unveil the crosstalk between the nuclear, chloroplast and mitochondrial genomes. Additionally, it has recently been demonstrated that the crosstalk between cellular redox homeostasis and epigenetic regulation occurs through the involvement of melatonin (Ahmad et al., 2023). The major roles of melatonin in scavenging cellular ROS, along with the signaling intermediates of different signal transduction warrants its significant contribution to varied abiotic stress tolerance mechanisms (Colombage et al., 2023). Further investigation may open up new redox and epigenetic control directions in response to multiple abiotic stress tolerance with the hormonal crosstalk in plants.

Studying plant responses to stress combinations is complex and costly, yet crucial for developing resilient crops in the face of climate change. The controlled variable approach, which isolates one stress factor while keeping others constant, has revealed how stress combinations can trigger unique molecular defenses in plants. Advances in genomic technologies, such as multi-omics approaches, have deepened our understanding of these responses, but translating laboratory findings to field environments remains challenging. Integrating lab and field research with multidisciplinary technologies like nanobiotechnology and genome editing offers promising avenues to enhance crop resilience by fine-tuning stress signaling pathways and physiological adaptations. To further strengthen research, it is essential to identify key survival thresholds and nodes in plants under stress combinations and systematically integrate existing data to uncover the mechanisms behind these responses. Developing efficient research methods and exploring how plants prioritize and adapt to multiple stresses will be critical as future climate conditions become more unpredictable. By leveraging advanced technologies like machine learning, gene editing, and remote sensing, and by breeding multi-resistant plant varieties, we can better equip crops to withstand the increasingly complex stress environments of the future, ensuring food security and sustainable agriculture.

## 9 Conclusion and future directions

The reactive oxygen species is inevitable in any stress events in plants. The ROS generation in response to multiple abiotic stress regulates the stress tolerance mechanism in plants like a two-edged sword. The excessive production of ROS leads to oxidative stress and damage to the plant’s cellular machinery. Alternatively, an effective scavenging system may detoxify the ROS and produce a balanced redox signature. This will lead to downstream signaling by activating stress-responsive transcription factors and gene activation. Epigenetic regulation, on the other hand, demonstrates significant controlling gateways for multiple abiotic stress tolerance mechanisms. The cross-talk between the ROS pathway and epigenetic control involves a complex network of molecular events, e.g., histone modification, DNA methylation, DNA acetylation, deacetylation, etc. These regulatory nexuses modulate the gene expression, physiological modulations, antioxidative defense, etc., to enable the plants to adapt towards multiple abiotic cues. This emerging field warrants many questions for future research to unravel specific interactions between ROS and epigenetic modification in the context of abiotic stress tolerance. Some of the relevant future directions are summarized below,

1. Further research is necessary to unveil the duality of ROS signaling in response to abiotic stress combinations. Any stress situations instigate cellular redox alterations. The efficient scavenging mechanism leads to the generation of “signature ROS”, which helps in many defense gene regulations. In response to abiotic stress, the cellular ROS is generated as a first line of defense signal, which in turn, after effective scavenging, leads to the “balanced ROS” helping in retrograde signaling. This duality of ROS signal in response to the multiple abiotic stress factors should be deciphered so that such signaling can be used effectively in sustainable stress management.
2. The synergy between epigenetic control and ROS should be decoded. Although the interplay between the ROS and epigenetic control has been established, more intriguing study is necessary to uncover the synergy ultimately.
3. Unveiling the cross-talk between ROS, epigenetic modification, and hormonal regulation at the molecular level can provide a better understanding of multi-stress tolerance strategies in crops.
4. The transgenerational impact of ROS-induced epigenetic modifications must be evaluated to develop a sustainable crop management system against multiple abiotic stresses. The epigenetic control and development of transgenerational memory are currently under scientific investigation. The epigenetic imprint developed under multiple abiotic stresses under a changing cellular redox milieu would provide significant gateways for future agricultural applications.
5. More emphasis is required to integrate advanced genome editing tools, e.g.), Clustered Regularly Interspaced Short Palindromic Repeats (CRISPR)/CRISPR-associated (Cas) technology to develop multiple abiotic stress-tolerant climate-smart crops.
6. The major challenge is judicially utilizing all the information obtained for crop genome tailoring to overcome the future environmental crisis. The compete gene expressional blue print under multiple abiotic stress responses is the prior requirement for the successful implication of site-specific gene editing technology.
